# Inhibition of Perforin-Mediated Neurotoxicity Attenuates Neurological Deficits After Ischemic Stroke

**DOI:** 10.3389/fncel.2021.664312

**Published:** 2021-06-28

**Authors:** Yuhualei Pan, Dan Tian, Huan Wang, Yushang Zhao, Chengjie Zhang, Song Wang, Dan Xie, Dong Zhang, Yanbing Zhu, Yongbo Zhang

**Affiliations:** ^1^Department of Neurology, Beijing Friendship Hospital, Capital Medical University, Beijing, China; ^2^Beijing Clinical Research Institute, Beijing, China; ^3^Immunology Research Center for Oral and Systemic Health, Beijing Friendship Hospital, Capital Medical University, Beijing, China

**Keywords:** microglia, perforin, immunity, ischemic stroke, cytotoxicity, neurogenesis, gliogenesis

## Abstract

Perforin-mediated cytotoxicity plays a crucial role in microbial defense, tumor surveillance, and primary autoimmune disorders. However, the contribution of the cytolytic protein perforin to ischemia-induced secondary tissue damage in the brain has not been fully investigated. Here, we examined the kinetics and subpopulations of perforin-positive cells and then evaluated the direct effects of perforin-mediated cytotoxicity on outcomes after ischemic stroke. Using flow cytometry, we showed that perforin^+^CD45^+^ immune cells could be detected at 12 h and that the percentage of these cells increased largely until on day 3 and then significantly declined on day 7. Surprisingly, the percentage of Perforin^+^CD45^+^ cells also unexpectedly increased from day 7 to day 14 after ischemic stroke in Perforin1-EGFP transgenic mice. Our results suggested that Perforin^+^CD45^+^ cells play vital roles in the ischemic brain at early and late stages and further suggested that Perforin^+^CD45^+^ cells are a heterogeneous population. Surprisingly, in addition to CD8^+^ T cells, NK cells, and NKT cells, central nervous system (CNS)-resident immune microglia, which are first triggered and activated within minutes after ischemic stroke in mice, also secreted perforin during ischemic brain injury. In our study, the percentage of perforin^+^ microglia increased from 12 h after ischemic stroke, increased largely until on day 3 after ischemic stroke, and then moderately declined from days 3 to 7. Intriguingly, the percentage of perforin^+^ microglia also dramatically increased from days 7 to 14 after ischemic stroke. Furthermore, compared with wild-type littermates, Perforin 1^–/–^ mice exhibited significant increases in the cerebral infarct volume, neurological deficits, and neurogenesis and inhibition of neurotoxic astrogliosis. Interestingly, the number of CD45^+^CD3^+^ T cells was significantly decreased in Perforin 1^–/–^ mice compared with their wild-type littermates, especially the number of γδ T cells. In addition, Perforin 1^–/–^ mice had lower levels of IL-17 than their wild-type littermates. Our results identified a critical function of perforin-mediated neurotoxicity in the ischemic brain, suggesting that targeting perforin-mediated neurotoxicity in brain-resident microglia and invading perforin^+^CD45^+^ immune cells may be a potential strategy for the treatment of ischemic stroke.

## Introduction

Ischemic stroke is the leading cause of death and disability in adults worldwide (Voskoboinik et al., 2015; Yan et al., 2015; Hankey, 2017; Stinear et al., 2020). In addition to early vascular recanalization, neuroinflammation has become increasingly recognized as playing a critical role in secondary brain damage after ischemic stroke (Klein et al., 2017; Mizuma and Yenari, 2017; Wang et al., 2020). Once ischemic stroke occurs, central nervous system (CNS)-resident immune cells, i.e., microglia, are first triggered and activated within minutes, and this microglial activation is followed by an influx of blood-borne immune cells (Zhou et al., 2020). Recent studies have suggested that blood-borne innate and adaptive immune cells may determine the outcome of stroke (Kleinschnitz et al., 2013; Ito et al., 2019; Kang et al., 2020). Innate and adaptive immune cells are activated and release proinflammatory molecules, which contribute to the worsening of brain injury, or exert a beneficial effect and promote tissue repair (Yan et al., 2015). Previous research has suggested that γδ T cells, as a major source of interleukin (IL)-17, aggravate ischemic brain injury (Shichita et al., 2009). A recent study implied that CD3^+^CD4^–^CD8^–^ T cells (double-negative T cells; DNTs) exacerbate ischemic brain injury through the tumor necrosis factor alpha (TNF-α) signaling pathway (Meng et al., 2019). Current evidence suggests that IL-10 and transforming growth factor-β (TGF-β) are anti-inflammatory cytokines mainly produced by microglia, regulatory T cells, and astrocytes, which are required for sensory motor recovery after ischemic stroke (Lu et al., 2005; Kleinschnitz et al., 2013; Mao et al., 2017). However, the direct cytotoxic effects of invading immune cells in the ischemic brain remain to be clarified. Thus far, whether microglia also contribute to functional recovery after ischemic stroke through direct cytotoxic effects and their underlying signaling mechanisms have not been clearly defined.

Recent studies have suggested that the direct cytotoxicity induced by immune cells plays an important pathophysiological role in inflammatory disorders (Voskoboinik et al., 2015). Cytotoxic immune cells have a defined property; they usually express and regulate the secretion of perforin and serine protease granzymes. Cytotoxic immune cells mainly include cytotoxic T lymphocytes (CTLs) and natural killer (NK) cells (Voskoboinik et al., 2015). Previous research has shown that allergen-specific CD8^+^ T cells relieve allergic airway inflammation through perforin (Enomoto et al., 2012). However, the role of cytotoxic immune cells in ischemic stroke is controversial. Previous studies have indicated that NK cells control CNS inflammation by killing proinflammatory microglia within minutes of ischemic stroke (Lunemann et al., 2008). In contrast, NK cells also accelerate neuronal death in the ischemic brain in the context of brain infarction (Gan et al., 2014). In addition, CD8^+^ T cells catalyze perforin-mediated neurotoxicity in experimental stroke and other inflammatory and degenerative brain disorders (Meuth et al., 2009; Miro-Mur et al., 2020). These seemingly opposing effects of perforin-mediated neurotoxicity highlight the need for studies focused on perforin-positive immune cell heterogeneity after ischemic stroke.

Here, we visualized and characterized the kinetics and types of perforin^+^CD45^+^ immune cells during different stages after experimental murine ischemic stroke using Perforin1-EGFP transgenic mice and further revealed that microglia and invading immune cells induced by perforin-mediated direct neurotoxicity accelerate ischemic lesion size and impair neurological outcomes in a Perforin 1 knockout mouse model of experimental stroke.

## Materials and Methods

### Animals

All animal experiments and experimental protocols were approved by the Institutional Animal Care and Ethics Committee of Beijing Friendship Hospital. The mice were maintained in a pathogen-free, temperature-controlled environment under a 12 h light/dark cycle at Beijing Friendship Hospital.

*Prf1-*knockout (*Prf1*^–/–^) mice were purchased from the Jackson Laboratory (stock number: 002407). Eight- to twelve-weeks-old male *Prf1^–/–^* mice and control wild-type (*WT*) littermates were used in this study.

*Prf1/IRES-EGFP-T2A-Cre* mice were generated by the Shanghai Model Organisms Center, Inc. The CRISPR target sequence (5′-GTTACCACACAGCCCCACTGCGG-3′) was selected for integration of the *IRES-EGFP-T2A-Cre* sequence just before the stop codon of Prf1. Cas9 mRNA and sgRNAs were prepared by *in vitro* transcription. The donor plasmid Prf1/IRES-EGFP-T2A-Cre contained the IRES sequence, EGFP, T2A sequence and nuclear translocation signal (NLS)-cre. The 3.2-kb 5′-arm (upstream just before the stop codon of *Prf1*) and the 3.1-kb 3′-arm (downstream of the stop codon) were cloned into this vector. The donor vector with Cas9 mRNA and sgRNAs was micro injected into C57BL/6J fertilized eggs. The positive F0 generation mice for homologous recombination were identified by long PCR. The PCR products were further confirmed by sequencing. The genotype of F1 generation *Prf1* knock in heterozygous mice were further identified by long PCR. Male mice aged 8–12 weeks were used in all of these experiments.

### Genotyping of the Animals

The method used to genotype Prf1-WT and Prf1-Knockout was the official protocol provided at https://www.jax.org/Protocol?stockNumber=002407&protocolID=22353. DNA for genotyping was extracted from tail snips by the alkali pyrolysis method. Prf1-e (IRES-EGFP-T2A-Cre) mice were genotyped by two pairs of primers (5′→3′ sequences, P1: CTACGGCTGGGATGATGACC; P2: AAGGT ACCCAGGAATCGGGA; P3: ACACCACAGCTACTGATGCC; and P4: AGACCCCTAGGAATGCTCGT). The P1 and P2 primers detected a 650 bp wild-type band, and the P3 and P4 primers detected a 568 bp mutant band. The mice for which only a 568 bp band was detected were considered mutant mice and were used in the subsequent experiment.

### Induction of Focal Cortical Ischemic Stroke in Mice

Focal cerebral ischemic stroke targeting the right sensorimotor cortex and mainly involving the barrel cortex was induced by occlusion of the distal branches of the middle cerebral artery (dMCAO). Sham surgery was performed in an identical manner as stroke surgery except for occlusion (Doyle et al., 2015; Wang et al., 2020). Anesthesia was induced with 2% isoflurane (#R510-22, RWD Life Science) and maintained during surgery using 1.5% isoflurane supplemented with regular air. Cortical ischemia was achieved by permanent occlusion of the distal branches of the right middle cerebral artery (dMCA), which supplies the sensorimotor cortex. In addition to dMCA occlusion, 7 min ligation of both common carotid arteries (CCAs) was performed. The animals were placed in the surgery room for at least 30 min to allow them to adapt to the environment. The body temperature of the animals was maintained at 37 ± 0.5°C during the surgery and for up to 2 h after the surgery. There were at least 6 mice in each group in each step of the experiment.

### 2,3,5-Triphenyl Tetrazolium Chloride (TTC) Staining

Three days after the onset of dMCAO, animals in the different groups were sacrificed for assessment of brain infarction. 2,3,5-Triphenyltetrazolium chloride (#T8877, Sigma) staining was used to visualize damaged/dead brain tissue as previously described. Brains were removed, placed in a brain matrix, and then sliced into 1 mm coronal sections. The slices were incubated in 2% TTC solution at 37°C for 10 min and then stored in 4% paraformaldehyde for 24 h. Digital images of the caudal aspect of each slice were obtained with a flatbed scanner. The infarct, ipsilateral hemisphere, and contralateral hemisphere areas were measured using ImageJ software (version 1.8.0). The indirect method (subtraction of the cortical volume of the residual right hemisphere from the cortical volume of the intact left hemisphere) was used to calculate the infarct volume. Infarct measurements were performed under double-blind conditions. There are 6–10 mice in each group.

### Behavioral Analysis

All animals were placed in the surgery room for at least 30 min to allow them to adapt to the environment to ensure the consistency of behavioral measurements. There are 6–10 mice in each group.

The adhesive-removal test is a sensitive method used to monitor the severity and reversal of sensorimotor deficits after cerebral focal ischemia in mice. A small round quarter-sized adhesive was fixed to the forepaws of each mouse. The time it took for the mouse to contact and remove the adhesive was recorded. The animals were trained to perform the task for 3 days prior to stroke induction. Animals that demonstrated an inability to perform the task were excluded from the formal experiments. We then carried out the adhesive removal task 1 day before stroke to establish baseline performance and then 1, 3, 7, and 14 days after stroke. Analysis of performance on the task was carried out by a blinded investigator. The mean amounts of time (seconds, averaged from three to four trials) required to contact and remove the stimuli from each paw were recorded. All testing trials were conducted during the daytime.

Whisker tests were performed as previously described (Wang et al., 2020). For the whisker-evoked forelimb placing test, the mice were gently held by their torsos, and the whiskers on one side of the face were brushed against the corner of a platform to elicit the same-side forelimb being placed on the platform. For the whisker-evoked cross-midline placing test, the mice were held by their torsos and rotated 45°. Then, the lower side of their whiskers was perpendicularly brushed against the surface edge of the corner platform to elicit the opposite-side (upper side) forelimb being placed upon the platform. If the mice placed their forelimb upon the desktop after whisker brushing, the trial was recorded as successful. The eyes of the mice were covered to avoid the effects of visual information on performance in the test. If the tested forelimb was placed on the platform after whisker touch, the trial was recorded as successful. If the mouse was motionless or shook but failed to place a limb on the platform, the trial was recorded as a failure. Trials in which the animal struggled while being held were not counted. Every mouse was trained for 10 trials per side per day for 3 days to allow them to habituate to the test before the stroke. The same protocol was performed 24 h after stroke. The experimenters were blinded to the grouping of the mice during all behavioral tests.

### Flow Cytometry

Infarcted brains were harvested after perfusion via apex cordis by phosphate-buffered saline and digested (0.1 mg/L collagenase IV, 1% calf serum, and phosphate-buffered saline) at 37°C for 40 min. The cell suspensions were then filtered and washed with MACS buffer (5 g/L bovine serum albumin, 2 mM EDTA, and phosphate-buffered saline).

The following primary antibodies were used: anti-CD24 (#130-103-371, clone: M1/69, Miltenyi), anti-GLAST (ACSA-1) (#130-098-803, clone: ACSA-1, Miltenyi), anti-CD133 (#141215, clone: 315-2C11, Biolegend), anti-CD45 (#103115, clone: 30-F11, Biolegend), anti-CD11b (#101257, clone: M1/70, Biolegend), anti-CD3 (#100219, clone: 17A2, Biolegend), anti-TCR γ/δ (#118108, clone: GL3, Biolegend), anti-NK-1.1(#108748, clone: PK136, Biolegend), anti-CD4 (#100428, clone: GK1.5, Biolegend), anti-CD8b (#126633, clone: YTS156.7.7, Biolegend), anti-CD8a (#100712, clone: 53-6.7, Biolegend), anti-Ly-6G (#127616, clone: 1A8, Biolegend), anti-NK-1.1 (#108708, clone: PK136, Biolegend), anti-TCR β (#109206, clone: H57-597, Biolegend), and anti-TCR γ/δ (#107512, clone: UC7-13D5, Biolegend). The cells were incubated with these antibodies at 4° in lucifuge for 15 min.

After stimulating the cells with Cell Activation Cocktail (#423303, Biolegend) for 5 h, we used the Cyto-Fast^TM^ Fix/Perm Buffer Set (#426803, Biolegend) to assess cytokine secretion. The cells were incubated with anti-IL-17A (#506904, clone: TC11-18H10.1, Biolegend) and anti-IFN-γ (#505836, clone: XMG1.2, Biolegend) antibodies in the dark at room temperature for 20 min. Then, all samples were resuspended and acquired on a FACS Aria II flow cytometer (BD Biosciences). Flow cytometry data were analyzed using FlowJo V10 (BD Bioscience). There are 6–10 mice in each group.

### Statistical Analysis

GraphPad Prism 8 (GraphPad Software, San Diego, CA, United States) was used for the statistical analysis. The values are expressed as the mean and min to max. Analysis of significant differences was performed using Student’s *t*-test. *P* < 0.05 was considered significant.

## Results

### Accumulation of Perforin^+^CD45^+^ Cells in the Ischemic Brain

**FIGURE 1:**
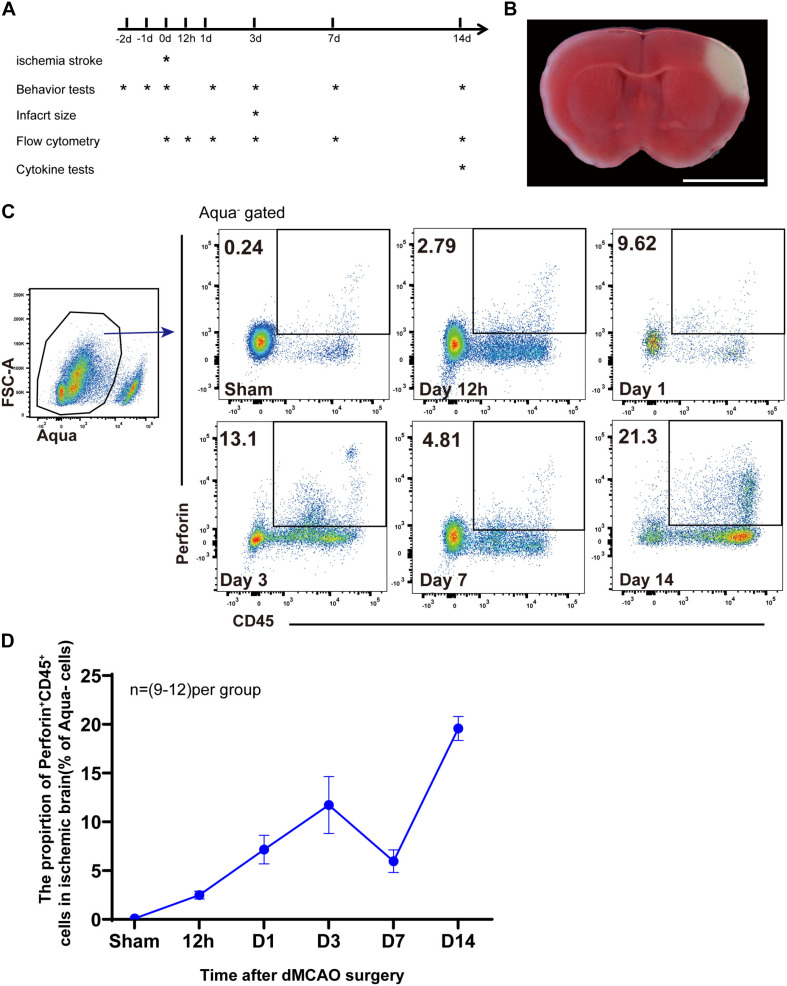
Accumulation of perforin^+^CD45^+^ cells in the ischemic brain. **(A)** Scheme for experimental design in a model of dMCAO. The timeline for outcome measurements is also illustrated. **(B)** Flat pattern of lateral ventricle cross-sections from mice subjected to dMCAO. Scale bar = 5 mm. **(C)** The proportion of live cells (FSC-A, Aqua^–^) expressing Prf1 (Prf1^–^GFP^+^CD45^+^) in the brain after dMCAO at six time points (sham, 12 h, day 1, day 3, day 7, and day 14) was assessed by flow cytometry. **(D)** The line chart shows the change in the number of Prf1-GFP^+^CD45^+^ cells at specific time points after surgery. The data are shown as the mean ± SEM; *n* = (9–12) mice per group.

We used experimental mouse models of distal middle cerebral artery occlusion (dMCAO) to examine the role of perforin-mediated neurotoxicity during ischemic stroke (Figures 1A,B). To investigate the kinetics of perforin-positive immune cells, i.e., Perforin^+^CD45^+^ cells, we induced dMCAO in Prf1-EGFP transgenic mice, in which a green fluorescent protein (GFP) reporter gene was integrated into the *prf1* allele. Representative plots are shown in Figure 1C. Our flow cytometry experiments showed that Perforin^+^CD45^+^ cells from the live cell gate (Aqua^–^) accumulated as early as 12 h after ischemic stroke and that the percentage of these cells increased largely until on day 3 and then significantly declined on day 7. Surprisingly, the percentage of Perforin^+^CD45^+^ cells once again increased and increased largely between days 7 and 14 after ischemic stroke (Figure 1D). These results indicated that Perforin^+^CD45^+^ cells play vital roles in the ischemic brain at early and late stages and further suggested that Perforin^+^CD45^+^ cells are a heterogeneous population.

### The Kinetics and Heterogeneity of Perforin^+^CD45^+^ Cells in the Ischemic Brain

**FIGURE 2:**
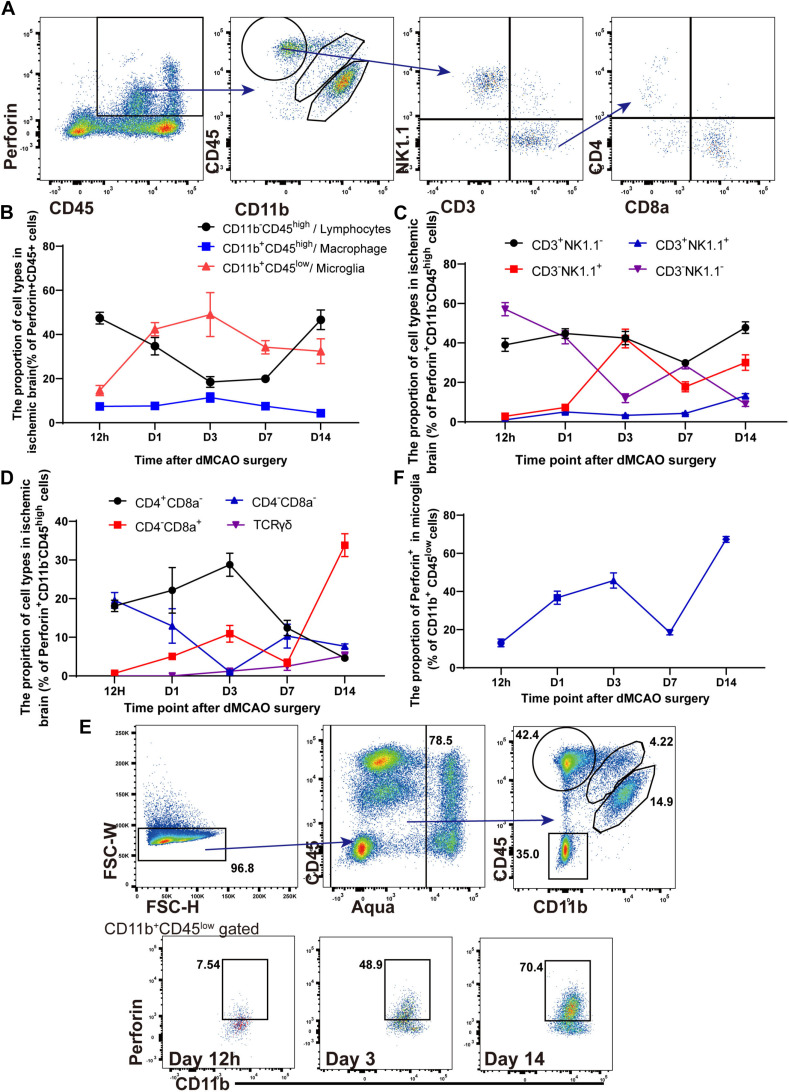
The kinetics and heterogeneity of Perforin^+^CD45^+^ cells in the ischemic brain. **(A)** The percentages of Perforin^+^CD11b^+^CD45^*low*^ microglia, Perforin^+^CD11b^+^CD45^*high*^ macrophages, and Perforin^+^CD11b^–^CD45^*high*^ lymphocytes in the ischemic brain were measured by flow cytometry. CD3^+^NK1.1^–^ cells was derived from perforin^+^CD11b^–^CD45^*high*^ lymphocytes and divided into CD4^+^ and CD8^+^ T cells. **(B)** The numbers of perforin^+^CD45^+^ lymphocytes, macrophages, and microglia after ischemic stroke at these time points. **(C)** The numbers of NK, NKT, CD3^–^NK1.1^–^, and CD3^+^NK1.1^+^ cells among perforin^+^CD11b^–^CD45^*high*^ lymphocytes after ischemic stroke at these time points. **(D)** The numbers of CD4^+^CD8^–^, CD4^–^CD8^+^, CD4^–^CD8^–^ and CD3^+^ TCRγδ^+^ cells among perforin^+^CD11b^–^CD45^*high*^ lymphocytes after ischemic stroke at these time points. **(E)** Flow cytometry showed the ability of Aqua^–^CD11b^+^CD45^*low*^ microglia to secrete perforin. **(F)** The proportion of Prf1-GFP among Aqua^–^CD11b^+^CD45^*low*^ microglia after dMCAO. The data are shown as the mean ± SEM; *n* = (6–10) mice per group.

Previous studies have suggested that NK cells, NK1.1^+^ NKT cells, CD4^+^ T cells, CD8^+^ T cells, and DNT cells mainly contribute to the secretion of perforin (Godfrey et al., 2015; Voskoboinik et al., 2015). However, there has been little research on the different subtypes of perforin^+^CD45^+^ cells after ischemic stroke. We identified the different subtypes of perforin^+^CD45^+^ cells in the ischemic brains of Prf1-EGFP transgenic mice from 12 h to 14 days after ischemic stroke. Representative plots of our data are shown in Figure 2A, and the flow cytometry results implied that Perforin^+^CD45^+^ cells comprised Perforin^+^CD11b^+^CD45^*low*^ microglia, Perforin^+^CD11b^+^CD45^*high*^ macrophages, and Perforin^+^CD11b^–^CD45^*high*^ lymphocytes in the ischemic brain. The percentage of Perforin^+^CD11b^+^CD45^*high*^ macrophages remained stable from 12 h to 14 days, and these cells accounted for only approximately 7.68% of Perforin^+^CD45^+^ cells (Figure 2B). In contrast, we found that the high percentage of perforin^+^CD11b^–^CD45^*high*^ lymphocytes accumulated at 12 h after ischemic stroke, evidently declined from 12 h to 7 days, and then dramatically increased from days 7 to 14 after dMCAO (Figure 2B). These findings provide a comprehensive characterization of the heterogeneity of perforin^+^ CD11b^–^CD45^*high*^ lymphocytes after dMCAO.

We next attempted to gain insight into the diversity of perforin^+^ CD11b^–^CD45^*high*^ lymphocytes during ischemic stroke. As shown in Figure 2C, we investigated the percentage of perforin^+^CD45^+^CD11b^–^ lymphocytes in the ischemic brain. Consistently, we detected prominent infiltration of different subtypes of perforin^+^CD45^+^CD11b^–^ lymphocytes, including CD3^–^NK1.1^+^ NK cells, CD3^–^NK1.1^–^ lymphocytes, CD3^+^NK1.1^+^ NKT cells, and CD3^+^NK1.1^–^ T cells into the ischemic brains of wild-type mice from 12 h to 14 days after dMCAO. The percentage of CD3^–^NK1.1^+^ NK cells gradually increased from 12 h to 3 days, increased largely on day 3, and then gradually decreased from days 3 to 14, which is consistent with previous work showing that the percentage of CD3^–^NK1.1^+^ NK cells increased largely on day 3 and then moderately declines after dMCAO (Gan et al., 2014). CD3^+^NK1.1^+^ NKT cells accumulated as early as 12 h after dMCAO, with the percentage of these cells slowly increasing from days 1 to 14 continuously after dMCAO. The percentage of CD3^–^NK1.1^–^ lymphocytes peaked 12 h after dMCAO and then evidently declined from 12 h to 14 days, with these cells accounting for only 1.058% of perforin^+^CD11b^–^CD45^+^ lymphocytes on day 14. The percentage of CD3^+^NK1.1^–^ T cells among perforin^+^CD11b^–^ CD45^*high*^ lymphocytes remained stable between 12 h and day 14. The CD3^+^NK1.1^–^ T cells mainly consisted of CD3^+^CD4^+^ T cells, CD3^+^CD8a^+^ T cells, DNT cells, and γδ T cells. In addition, previous works suggested that CD3^+^CD4^+^ T cells, CD3^+^CD8a^+^ T cells, DNT cells, and γδ T cells exhibit diverse functions in ischemic stroke by regulating immunological and inflammatory homeostasis (Gill and Veltkamp, 2016; Ito et al., 2019; Meng et al., 2019). Next, we further comprehensively analyzed the different subtypes and of CD3^+^NK1.1^–^ T cells and the percentages of these subtypes in the ischemic brain (Figures 2A,D).

In our study, similarly, CD3^+^CD4^+^ T cells and DNT cells were detected as early as 12 h, and the percentage of DNT cells then evidently declined from 12 h to 14 days, with these cells accounting for only 7.698% of CD3^+^NK1.1^–^ T cells on day 14 in the ischemic brain. In contrast, the percentage of CD3^+^CD4^+^ T cells slowly increased and peaked on day 3 and then declined from day 3 to day 14 after dMCAO (Figure 2D). Moreover, our experiments showed that the percentage of γδ T cells slowly increased until on day 14 after dMCAO, which is consistent with previous research showing that γδ T cells accelerate brain injury in the delayed phase of dMCAO (Shichita et al., 2009). Furthermore, kinetic experiments showed that CD3^+^CD8a^+^ T cells were detected as early as 12 h and that the percentage of these cells remained stable from 12 h to 7 days and then dramatically increased cotinuously on day 14 after dMCAO.

Collectively, these findings suggested that infiltrating CD3^–^NK1.1^+^ NK cells, CD3^–^NK1.1^–^ lymphocytes, and CD3^+^NK1.1^+^ NKT cells play a vital role from 12 h to 7 days after dMCAO. However, CD3^+^CD8a^+^ T cells and γδ T cells contribute to the secretion of perforin during the delayed phase of ischemic stroke.

Microglia are activated within minutes after ischemic stroke (Davalos et al., 2005). Surprisingly, as shown in Figures 2B,E,F, the percentage of perforin^+^CD11b^+^CD45^*low*^ microglia also increased from 12 h to 3 days, gradually decreased and then remained stable from days 3 to 14. Furthermore, CD11b^+^CD45^*low*^ microglia accounted for 14.612–49.3% of perforin^+^CD45^+^ immune cells from 12 h to 3 days, when a pronounced inflammatory response occurred. Furthermore, our results showed that, the percentage of perforin^+^ microglia among CD11b^+^CD45^*low*^ microglia increased from 12 h after dMCAO, peaked on day 3 after dMCAO, and then moderately declined from days 3 to 7. Intriguingly, the percentage of perforin^+^ microglia also dramatically increased from days 7 to 14 after dMCAO (Figures 2E,F), and the percentage of perforin^+^ microglia cells reached 67.32 ± 1.521% on day 14. Our results suggested that in addition to secreting inflammatory molecules, microglia are also along with other types of perforin^+^CD45^+^ cells are major players, possibly interacting with other cells in the ischemic brain through a direct perforin-mediated cytotoxic pathway.

### Perforin^+^CD45^+^ Cells Significantly Worsen Outcomes After Ischemic Stroke

**FIGURE 3:**
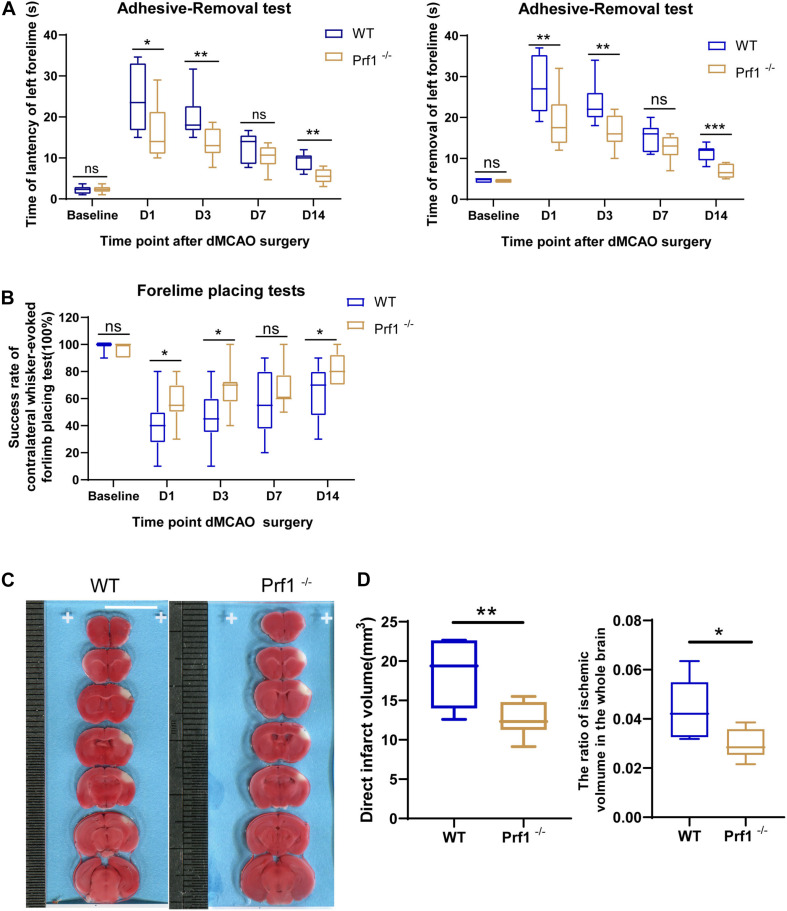
Perforin^+^CD45^+^ cells significantly worsen outcomes after ischemic stroke. **(A)** The adhesive removal test was performed to examine the effect of *Prf1*^–/–^ on functional recovery (latency and removal time) before surgery and 12 h, 1, 3, 7, and 14 days after focal cerebral ischemia. *n* = (6–10) animals in each group; *Prf1*^–/–^ mice vs. *WT* mice at each time point. *P < 0.05, **P < 0.01, ***P < 0.001. **(B)** There was a significant difference in performance on the whisker test between *Prf1*^–/–^ and *WT* mice at the time points mentioned before. **(C)** TTC-stained sections from adult *WT* and *Prf1*^–/–^ mice 3 days after dMCAO. Scale bars = 10 mm. **(D)** The box plot summarizes the infarct volume and infarct volume ratio data. The data are shown as the median and the value of min to max; *n* = (6–10) animals in each group. *P < 0.05, **P < 0.01, ***P < 0.001.

To further examine how perforin-mediated neurotoxicity affects outcomes after ischemic stroke, *Prf1^–/–^* mice were subjected to dMCAO to further confirm the critical role of the direct cytotoxic effects of invading immune cells and CNS-resident immune cells, i.e., microglia, on the ischemic brain. As expected, in the adhesive removal test (Figure 3A), the time required to contact or remove the adhesive dot from the left paw was significantly decreased in *Prf1^–/–^* mice compared to *WT* mice. The whisker test (Figure 3B) was also performed at the same time points. *Prf1^–/–^* mice exhibited better whisker trigger motion sensing than *WT* mice. TTC staining revealed that the infarct volume (Figures 3C,D) was significantly decreased in *Prf1^–/–^* mice compared to *WT* mice. In short, these results suggested that perforin is an important factor in worsening brain-related outcomes after ischemic stroke.

### Inhibition of Perforin-Mediated Neurotoxicity Facilitates Neurogenesis

**FIGURE 4:**
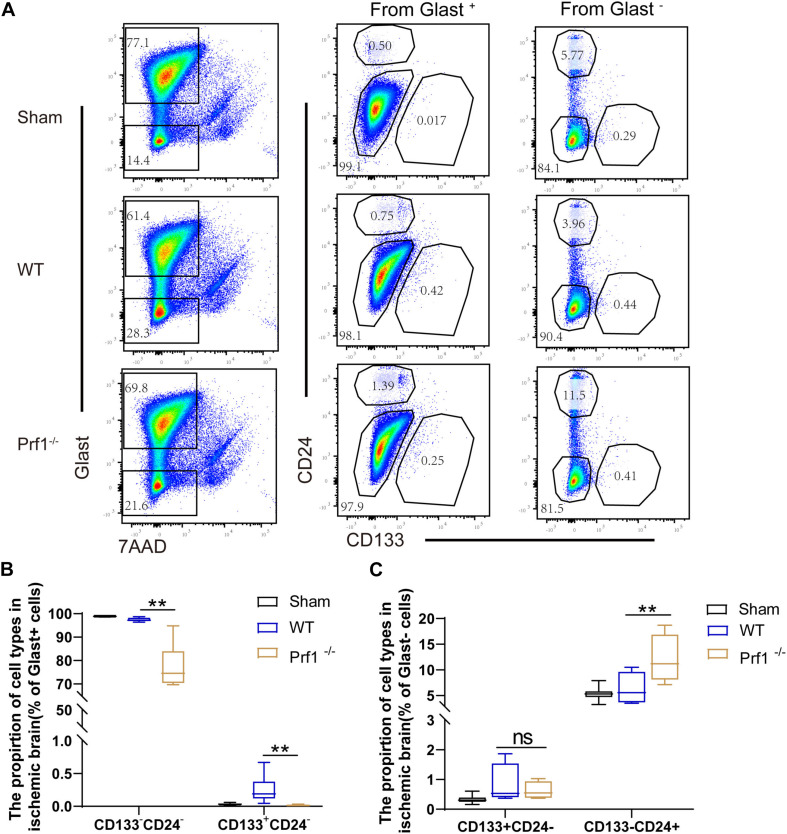
Inhibition of perforin-mediated neurotoxicity facilitates neurogenesis. **(A)** Flow cytometry was used to detect astrocytes and neuron stem/progenitor cells by according to Glast and 7AAD expression, and these cells were divided into Glast^+^ and Glast^–^ subtypes 14 days after ischemia surgery and sham operation group in the focal ischemic brain area. Then, these three subtypes were divided into stem/progenitor cells according to CD133 and CD24 expression. **(B,C)** The box graph shows the numbers of mature astrocytes and glial progenitors. The data are shown as the median and the value of min to max; *n* = (6–10) animals in each group. *Prf1*^–/–^ mice vs. *WT* mice at each time point. **P < 0.01.

Previous studies have suggested that activated CD8^+^ T lymphocytes inhibit neural stem/progenitor cell proliferation via interferon-gamma (IFN-γ) and that activated regulatory T cells increase neurogenesis through IL-10 and TGF-β after ischemic stroke (Guo et al., 2010; Hu et al., 2014). To determine whether perforin regulates neurogenesis after ischemic stroke, we collected neural stem/progenitor cells from *Prf1^–/–^* mice on day 14 after dMCAO. Representative plots are shown in Figure 4A. Briefly, neural stem/progenitor cells were isolated from the ischemic brain according to Glast, CD24 and CD133 expression (Liu et al., 2016; Jin et al., 2020). From the Glast^+^ pool, we isolated Glast^+^CD133^+^CD24^–^ neural stem/progenitor cells and other Glast^+^CD133^–^CD24^–^ astrocytes. Finally, from the Glast^–^ cell fraction, Glast^–^CD133^+^CD24^–^ neural stem/progenitor cells and Glast^–^CD133^–^CD24^+^ neuroblasts were isolated. Flow cytometry experiments showed that the percentage of Glast^+^CD133^+^CD24^–^ glial stem/progenitor cells and Glast^+^CD133^–^CD24^–^ astrocytes decreased in *Prf1^–/–^* dMCAO mice. However, the percentage of Glast^–^CD133^–^CD24^+^ neuroblasts increased in *Prf1^–/–^* dMCAO mice, suggesting that perforin-mediated neurotoxicity impaired neurogenesis and promoted neurotoxic astrogliosis after ischemic stroke (Figures 4B,C).

### Inhibition of Perforin Impairs the Infiltration of CD3^+^NK1.1^–^ T Cells and γδ T Cells

**FIGURE 5:**
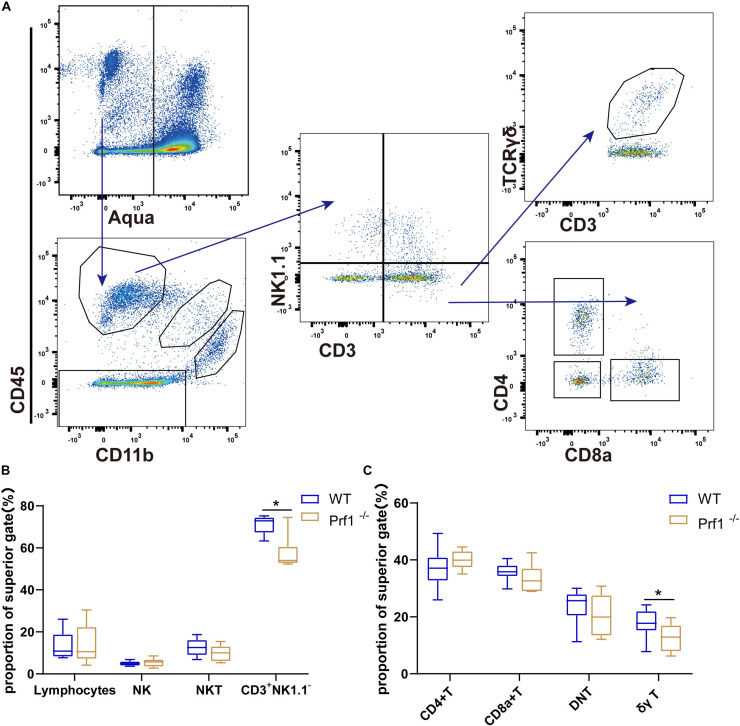
Inhibition of perforin impairs the infiltration of CD3^+^NK1.1^–^ T cells and γδ T cells. **(A)** Flow diagram showing various lymphocyte subtypes. Lymphocytes are Aqua^–^CD11b^–^CD45^+^ cells, and they can be divided into several cell subtypes according to the expression of classical markers, such as CD3, NK1.1, TCRγδ, CD4, and CD8. **(B,C)** The plot displays the proportions of these lymphocytes. The data are shown as the median and the value of min to max; *n* = (6–10) animals in each group. *Prf1*^–/–^ mice vs. *WT* mice at each time point. *P < 0.05, **P < 0.01.

A critical question is the mechanism by which perforin inhibits neurorepair during ischemic stroke. Representative plots are shown in Figure 5A and Supplementary Figure 1A. Flow cytometry was used to analyze the percentages and types of CD45^+^CD3^+^ T cells after ischemic stroke in *Prf1^–/–^* mice on at day 14 after dMCAO. Sham operation group shows few CD11b^–^CD45^*high*^ lymphocytes except CD11b^–^CD45^*low*^ microglia cells (Supplementary Figure 1A). Our results showed that the percentage of CD3^+^ NK1.1^–^ T cells decreased in the ischemic brain in *Prf1^–/–^* mice on day 14 after dMCAO, especially the percentage of proinflammatory γδ T cells (Figures 5B,C). Our results further suggested that, in addition to directly killing hypoxic neurons, perforin directly or indirectly cooperates with invading immune cells and CNS-resident immune microglia during ischemic brain injury.

### Inhibition of Perforin Modulates the Secretion of IFN-γ and IL-17 After Ischemic Stroke

**FIGURE 6:**
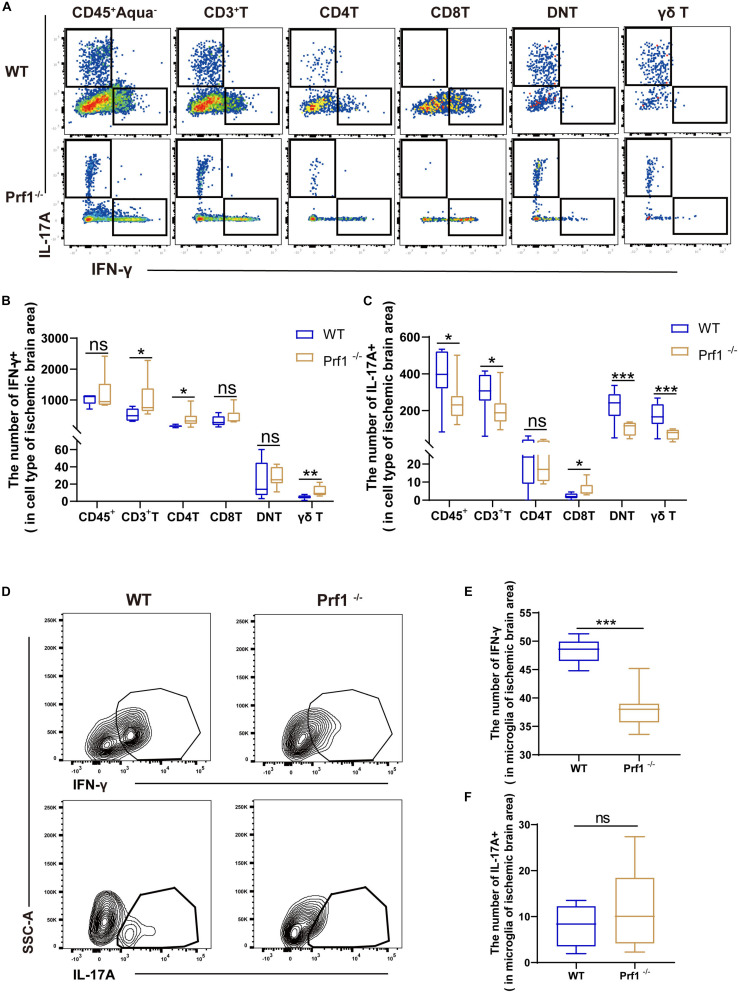
Inhibition of perforin modulates the secretion of IFN-γ and IL-17 after ischemic stroke **(A)** The pseudocolor flow plot reveals the expression of IFN-γ and IL-17 in the ischemic brain area 14 days after dMCAO. **(B,C)** This statistical chart shows the expression levels of IL-17A or IFN-γ in various cells. **(D)** The contour map shows the secretion of IFN-γ and IL-17 from CD11b + CD45^*low*^ microglia in *Prf1*^–/–^ mice compared to *WT* mice. **(E,F)** The box chart shows the differences in cytokine secretion from microglia between *Prf1*^–/–^ and wild-type mice after dMCAO. The data are shown as the median and the value of min to max; *n* = (6–10) animals in each group. *Prf1*^–/–^ mice vs. *WT* mice at each time point. *P < 0.05, **P < 0.01, ***P < 0.001.

Previous studies have asserted that IFN-γ and IL-17 augment the lesion size and inhibit neurogenesis in mice subjected to dMCAO (Meuth et al., 2009; Shichita et al., 2009; Zhang and Chopp, 2016; Lin et al., 2019). We identified that perforin^+^CD45^+^CD3^+^ cells are a major source of IFN-γ and IL-17 (Supplementary Figures 2A,B), which may boost local inflammation, as demonstrated above. Representative plots of our data are shown in Figures 6A,D. Flow cytometry analysis revealed that the numbers of DNT cells and γδ T cells expressing IL-17 were greatly reduced in *Prf1^–/–^* mice subjected to dMCAO (Figures 6A,B) and that the number of microglia expressing IFN-γ was greatly reduced in *Prf1^–/–^* mice subjected to dMCAO (Figures 6D,E). In contrast, we found that the numbers of CD4^+^ T cells expressing IFN-γ and microglia expressing IL-17 were increased in *Prf1^–/–^* mice subjected to dMCAO (Figures 6C,F). These findings partly implied that perforin might modulate the production of IFN-γ and IL-17.

## Discussion

The major findings of this study are (1) the kinetics of perforin^+^CD45^+^ cells invading the ischemic area, (2) the fact that perforin exerts a deleterious impact after ischemic stroke, and (3) the fact that perforin-mediated direct neurotoxicity controls the number of infiltrating γδ T cells and may further increase the secretion of the proinflammatory cytokines IL-17 and IFN-γ. As documented here using Prf1-EGFP transgenic mice, the kinetics of perforin mainly induced direct cytotoxic effects of brain-resident microglia and invading immune cells, and perforin did not have an effect on stroke outcomes in *Prf1^–/–^* mice subjected to dMCAO; however, the neurological deficits and histological outcomes paralleled the change in neurogenesis. Our findings unequivocally supported the hypothesis that perforin plays a critical role in delaying neurorepair.

The perforin-mediated direct cytotoxic pathway is shared by cytotoxic lymphocytes, which mainly include cytotoxic T lymphocytes (CTLs), NKT cells, NK cells, and other DNT cells (Lunemann et al., 2008; Voskoboinik et al., 2015). Despite substantial differences in how these different cell types are activated and how they recognize their targets, the key pathways that mediate target cell death are conserved. Nevertheless, the cellular and molecular interactions of various types of perforin^+^CD45^+^ T cells with other cells in the ischemic brain that ultimately determine the outcome after ischemic stroke are controversial. For example, as a first line of defense, NK cells contribute to the genesis of brain lesions at the very initiation of stroke (Gan et al., 2014). In contrast, some studies have shown that NK cells do not worsen outcomes after ischemic stroke (Gan et al., 2014; Earls et al., 2020; Miro-Mur et al., 2020).

Thus, this discrepancy points to several potentially critical issues that need to be considered. First, what kinetics or time window of perforin -positive immune cells invading the brain are underestimated after ischemic stroke? Our kinetics experiments showed that CD3^–^NK1.1^–^ immune cells accumulated and that the percentage of these cells peaked as early as 12 h after dMCAO, with these cells accounting for 57.08% of perforin^+^ lymphocytes. In addition, release of granzyme B (GZMB) by helper CD4^+^ T cells causes glial fibrillary acidic protein (GFAP) fragmentation in astrocytes (Stopnicki et al., 2019). Whether CD4^+^ T cells expressing perforin worsen outcomes after dMCAO needs to be further investigated. Future studies should determine whether and when other types of bona fide immune cells contribute to the pathogenesis of ischemic stroke.

Moreover, do perforin^+^CD45^+^ T cells directly or indirectly coordinate with immune cells or brain-resident microglia in the ischemic brain? For example, NK cells might cooperate with monocytes and platelets to propagate thrombosis and activate the complement system after ischemic stroke (Spahn et al., 2014; Chen et al., 2019; Wang et al., 2020). Interestingly, the number of CD8^+^ T cells is increased in animals with NK1.1^+^ NK cell depletion compared with control animals 7 days after ischemia onset (Miro-Mur et al., 2020), which is reminiscent of our results showing that perforin inhibits the infiltration of CD45^+^CD3^+^ T cells, especially decreasing the percentage of proinflammatory γδ T cells in *Prf1^–/–^* mice subjected to dMCAO.

Microglia in the brain are mononuclear phagocytes that are increasingly being recognized to be essential players in the homeostasis, development, and diseases of the CNS (Salter and Stevens, 2017; Madore et al., 2020). Previous works have shown that brain-resident microglia contribute to the “first line of defense” after ischemic stroke, can be activated within minutes by danger-associated molecules and release multiple proinflammatory molecules and anti-inflammatory cytokines (Yan et al., 2015; Filipello et al., 2018; Qin et al., 2019). However, the direct impact of microglia on the effects of the perforin-mediated cytotoxic pathway after ischemic stroke has not been previously uncovered. Intriguingly, our results suggested that microglia secrete high levels of perforin across the stages of ischemic stroke and play deleterious roles through contact-dependent perforin-mediated direct cytotoxicity. Previous works have suggested that CD8 + T cells contribute to neuronal damage by antigen-dependent perforin-mediated electrical silencing of neurons and that NK cells accelerate brain infarction because ischemic neurons lose their self-identity, modulating NK cell tolerance and then activating NK cells (Meuth et al., 2009; Miro-Mur et al., 2020). However, the exact mechanism by which microglia accelerate brain infarction by perforin-mediated direct cytotoxicity remains to be elucidated.

The adult mammalian brain contains a population of NSCs in the subventricular zone (SVZ) and sub-granular zone (SGZ) of the dentate gyrus (Denoth-Lippuner and Jessberger, 2021). NSCs are multipotent cells and give rise to neuroblasts, which migrate to the lesion site and differentiate into new neurons. Neurogenesis is thought to be a key process to promote post-stroke recovery and repair (Rahman et al., 2021). However, recent works suggested that NSCs also give rise to a subpopulation of reactive astrocytes in the lesion site that contribute to astrogliosis and scar formation, which inhibit the post-stroke recovery and repair (Shimada et al., 2012; Faiz et al., 2015; Ito et al., 2019). That is, NSCs are divided into neural stem/progenitor cells and glial stem/progenitor cells, which could differently proliferate and differentiate into neuroblasts and reactive astrocytes. There was a complex interplay between neuroinflammation and neurogenesis and astrogliosis that modulate ischemic stroke outcome and possibly recovery. Recent studies have indicated that migrating cells are capable of imparting immunomodulatory effects that can influence the proliferation and differentiation of NSCs (Dulken et al., 2019; Vicidomini et al., 2020).

Moreover, T cells can inhibit the proliferation of NSCs in part by secreting IFN-γ and IL-17 (Li et al., 2013; Zhang and Chopp, 2016; Wang et al., 2017; Chen et al., 2020). Our data demonstrated that the percentage of Glast^+^CD133^+^CD24^–^ glial stem/progenitor cells and Glast^+^CD133^–^CD24^–^ astrocytes decreased and the percentage of Glast^–^CD133^–^CD24^+^ neuroblasts increased in *Prf1^–/–^* dMCAO mice. In addition, there was no difference on percentage of Glast^–^CD133^+^CD24^–^ neural stem/progenitor cells between wild-type mice and *Prf1^–/–^* dMCAO mice. The main reasons were that inhibition of perforin might promote neural stem/progenitor cells differentiate into neuroblasts. Here, we suggested that perforin-mediated neurotoxicity impaired neurogenesis and promoted neurotoxic astrogliosis after ischemic stroke. Although the exact mechanism of the interactions between perforin and neurogenesis remains to be elucidated, our results provide a possible cause for the decline in neurogenesis during ischemic stroke.

## Conclusion

In conclusion, our study provides important new insights into the mechanisms of perforin-mediated direct cytotoxicity after ischemic stroke.

## Data Availability Statement

The raw data supporting the conclusions of this article will be made available by the authors, without undue reservation.

## Ethics Statement

The animal study was reviewed and approved by the Institutional Animal Care and Ethics Committee of Beijing Friendship Hospital, Capital Medical University.

## Author Contributions

YP was participated in performing the research, analyzing the data, and initiating the original draft of the article. DT, HW, YZhao, CZ, SW, DX, and DZ were also participated in performing the research and collecting the data. YZhu and YZhang established the hypotheses, supervised the studies, analyzed the data, and co-wrote the manuscript. All authors participated meaningfully in the study and have read and approved the submission of this manuscript.

## Conflict of Interest

The authors declare that the research was conducted in the absence of any commercial or financial relationships that could be construed as a potential conflict of interest.

## Supplementary Material

Supplementary Figure 1 Mice without induced dMCAO model have few CD11b^–^CD45^+^ lymphocytes in their brains. **(A)** A flow cytometry diagram showed that the brain cells in the sham group were screened by CD45 and CD11b antibodies after removing the dead cells, and there were few lymphocytes except microglia cells.

Supplementary Figure 2 Perforin^+^CD45^+^ cells secreted IFN-γ and IL-17. **(A)** Flow diagram showing the gating strategy of Perforin^+^CD45^+^ cells and their subsets. **(B)** The cytokine secretion of Perforin^+^CD45^+^ cells and their subsets were shown by histogram. **(C)** This statistical chart shows the number of IL-17A or IFN-γ in Perforin^+^CD45^+^ and their subsets cells in ischemic stroke area at day 14. The data are shown as the median and the value of min to max; n = 8 animals in each group.
