# Supplementary figures and images for: Inhibition of Perforin-Mediated Neurotoxicity Attenuates Neurological Deficits After Ischemic Stroke
Source: Front Cell Neurosci. 2021 Jun 28;15:664312. doi: 10.3389/fncel.2021.664312 (PMC8274971; doi:10.3389/fncel.2021.664312)

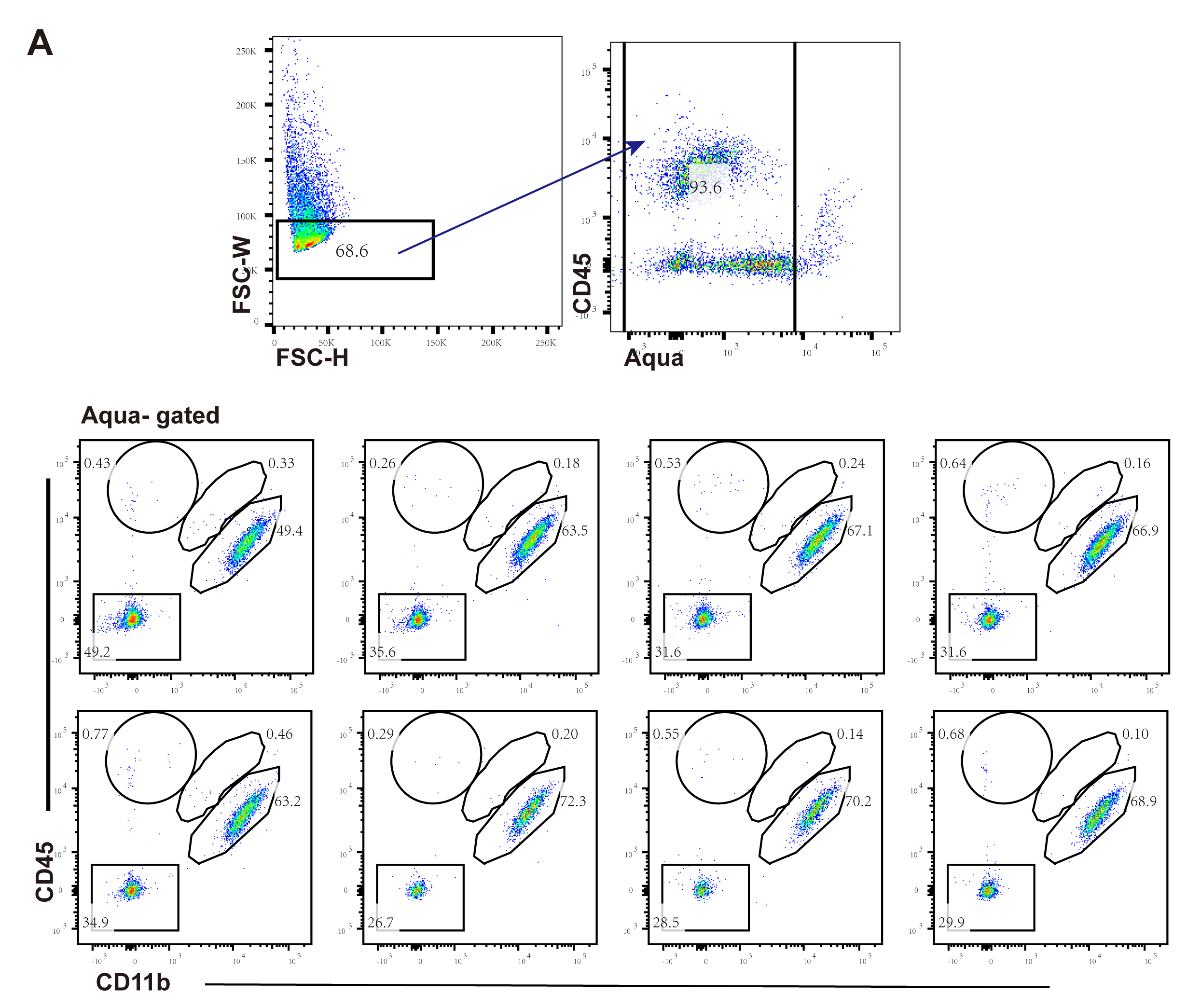

Supplement: Supplementary Figure 1 — Mice without induced dMCAO model have few CD11b–CD45+ lymphocytes in their brains. (A) A flow cytometry diagram showed that the brain cells in the sham group were screened by CD45 and CD11b antibodies after removing the dead cells, and there were few lymphocytes except microglia cells. [file Image_1.TIF]

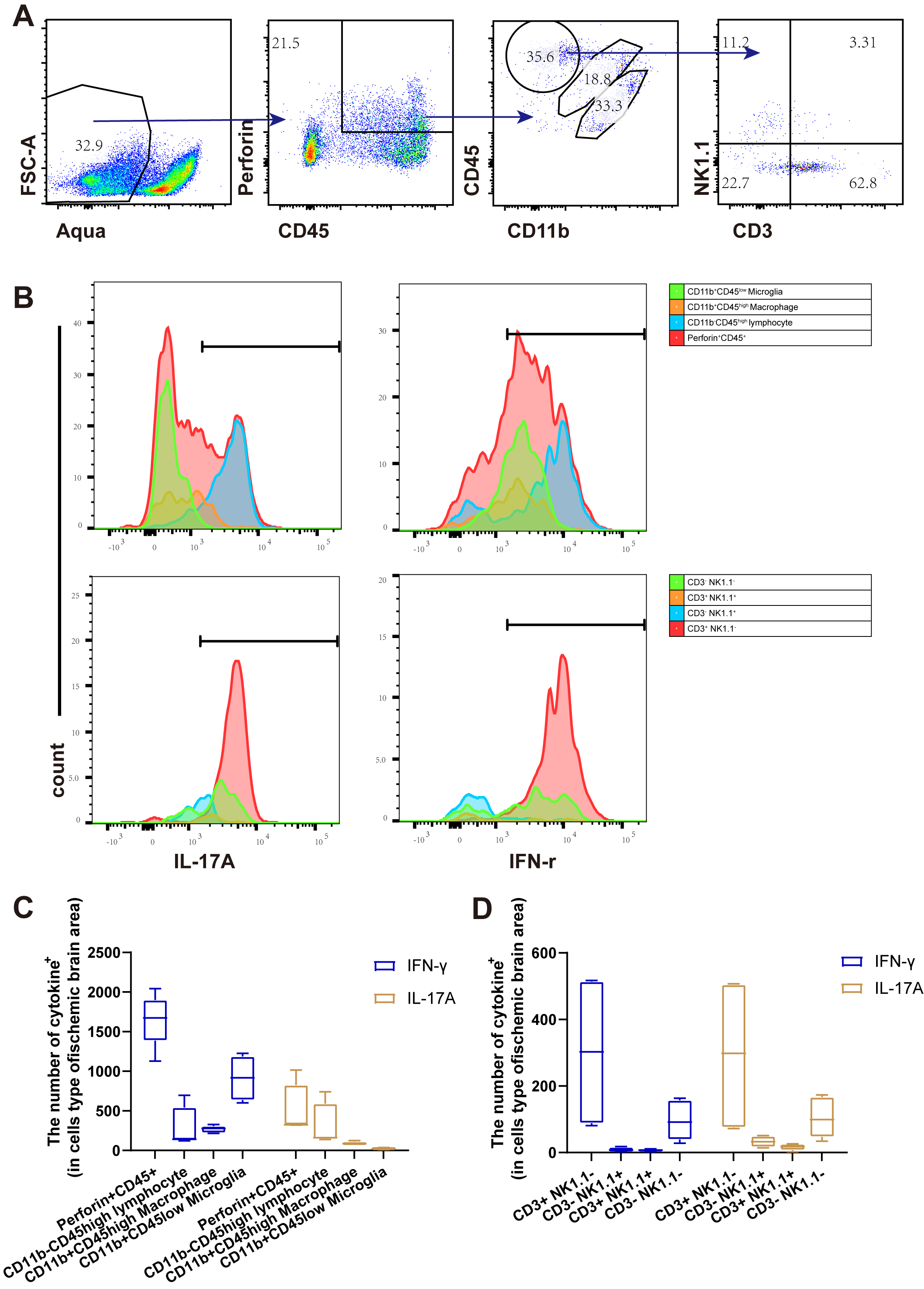

Supplement: Supplementary Figure 2 — Perforin+CD45+ cells secreted IFN-γ and IL-17. (A) Flow diagram showing the gating strategy of Perforin+CD45+ cells and their subsets. (B) The cytokine secretion of Perforin+CD45+ cells and their subsets were shown by histogram. (C) This statistical chart shows the number of IL-17A or IFN-γ in Perforin+CD45+ and their subsets cells in ischemic stroke area at day 14. The data are shown as the median and the value of min to max; n = 8 animals in each group. [file Image_2.TIF]
